# *In vitro* activity of *Erythrophleum ivorense* extract against the promastigote stage of cutaneous *Leishmania* parasite, a member of *Leishmania (Mundinia) enriettii* complex isolates from Ghana

**DOI:** 10.1099/acmi.0.000050

**Published:** 2019-09-03

**Authors:** Alberta Serwah Anning, Godwin Kwakye-Nuako, Elvis Ofori Ameyaw, Mba-Tihssommah Mosore, Kwame Kumi Asare

**Affiliations:** ^1^​ Department of Biomedical Sciences, School of Allied Health Sciences, College of Health and Allied Sciences, University of Cape Coast, Cape Coast, Ghana; ^2^​ U. S. Naval Medical Research Unit No. 3 (NAMRU-3), Noguchi Memorial Institute for Medical Research, University of Ghana, Legon, Accra, Ghana; ^3^​ Department of Parasitology, Noguchi Memorial Institute for Medical Research, University of Ghana, Legon, Accra, Ghana; ^4^​ Department of Protozoology Institute of Tropical Medicine (NEKKEN), Nagasaki University Sakamoto 1-12-4, Nagasaki 852-8523, Japan

**Keywords:** Cutaneous leishmaniasis, *Leishmania enriettii* complex, leishmanicidal, *Erythrophleum ivorense*, promastigotes stage, Ghana

## Abstract

**Background:**

Cutaneous leishmaniasis causes physical disfigurement and impairment on affected individuals, however, little attention has been paid to it eradication. The situation of this neglected disease is complicated with the expansion of the non-human pathogenic *Leishmania enriettii* complex causing infection in humans. We have previously shown that the extract from *Erythrophleum ivorense* has leishmanicidal activity against promastigote stages of the *L. enriettii* complex isolate from Ghana and *L* *eishmania* *donovani*. The extract of *E. ivorense* has shown to have anti-inflammatory, wound-healing ability, antiallergic, antimalarial and antischistosomal activity. However, the concentration threshold of *E. ivorense* extract required for leishmanicidal activity against the emerging human pathogenic *L. enriettii* complex isolates is not clear.

**Aim:**

To test for the concentration threshold of *E. ivorense* extract required to obtain ideal leishmanicidal activity against the promastigote stage of human pathogenic *L. enriettii* complex isolates from Ghana.

**Method:**

The ethanolic leaf extract of *E. ivorense* was serially diluted and tested against the promastigote stage of the *L. enriettii* complex. Parasite inhibition was measured at 590 nm using a spectrophotometer after staining parasites with trypan blue. To select the threshold concentration for maximum inhibition of the promastigote stage of the *L. enriettii* complex, the concentration cut-off statistic was used.

**Results:**

The MIC of *E. ivorense* extract for *L. enriettii* promastigote inhibition was 62.3 μg ml^−1^. The highest promastigote inhibition was observed at 72 h.

**Conclusion:**

We show that a MIC of 62.3 μg ml^−1^ of *E. ivorense* leaf extract exhibits an ideal leishmanicidal activity against the promastigote stage of *L. enriettii* complex isolates.

## Introduction

The therapeutic value of medicinal plants cannot be overemphasized [1]. Therapeutic activities of medicinal plants against infection-causing pathogens and immune-mediated diseases are widely known [2–4]. *Cinchona officinalis* and *Artemisia annua* medicinal plants that produce quinine and artemisinin, respectively, are currently the most effective antiplasmodial drugs [5, 6]. Similarly, the extracts from aerial parts of *Erythrophleum ivorense* have shown to pharmacologically contain antiallergic, antioxidant, antihypertension, antitumour, anti-inflammatory, antiviral, hypo-lipidemic, antiarrhythmic, antimalarial and anticancer properties [7, 8]. The leaf extracts of *E. ivorense* have been reported to also have antischistosomal activity [9]. *E. ivorense* is a major medicinal plant for the treatment of smallpox, inflammation, wound healing and convulsion in Ghana [10, 11] and with other several medicinal uses throughout Africa [12]. Recently, we showed that leaf extract of *E. ivorense* has high cytotoxicity against the promastigote stage of cutaneous *Leishmania* species, *L. enriettii* complex isolates from Ghana [12, 13].

Leishmaniasis remains one of the most neglected diseases caused by kinetoplastid protozoan *Leishmania* [14, 15]. Leishmaniasis is a spectrum of diseases that presents as cutaneous, mucocutaneous and visceral forms that infect a wide range of specific hosts [16, 17]. Nearly 4 million new cases of leishmaniasis occur every year with about 70 000 related deaths [18]. The expansion and cross-host infections by zoonotic *Leishmania* species is a cause of concern to the already challenging treatment and clinical management of the disease [19]. Current treatment efficacy varies substantially from species or geographical isolates [20, 21].

A non-human pathogenic *Leishmania* parasite, *L. enriettii* complex, which causes cutaneous leishmaniasis in guinea pigs (*Cavia porcellus*), red kangaroo (*Macrofus rufus*), northern wallaroos (*Macropus robustus woodwardii*), black wallaroos (*Macropus bernardus*) and agile wallabies (*Macropus agilis*), recently has been isolated from human infections in Martinique Island, Thailand and Ghana [22–24]. The ability of the *L. enriettii* complex, a causative agent of cutaneous leishmaniasis (CL) to infect a wide range of vertebrate hosts suggests its high plasticity [25, 26]. However, many aspects of this *Leishmania* parasite including its biology, vectorial transmission and epidemiology are still unknown.

In 2015, this Ghanaian *Leishmania* isolate was identified to belong to the *L. enriettii* complex [24]. Recent research has intimated the possibility of adapting the Ghanaian isolate into a laboratory guinea-pig model to study the infective amastigote stage.

This study was therefore conducted to identify the MIC of *E. ivorense* leaf extract required to obtain ideal leishmanicidal activity against the promastigote stage of human pathogenic *L. enriettii* complex isolates from Ghana. We show that the minimum concentration of the leaf extract of *E. ivorense* for ideal leishmanicidal activity is 62.5 μg ml^−1^.

## Methods

### Plant extracts

*E. ivorense* (Fabaceae) leaves were collected from Cape Coast in the Central Region of Ghana between April 2012 and August 2013. The plants were authenticated by Botanists in the School of Biological Sciences. The leaves of *E. ivorense* [BHM/Eryth/017R/2014], were crude extracted using a previously described method [12]. The samples were pulverized and crude compounds extracted using 70 % ethanol in round bottom flasks for 3 continuous days. The ethanol was decanted from the extracts and further filtered using Whatman filter paper. The filtrates were concentrated to a semi-solid extract with a rotary evaporator regulated at 60 °C and completely dried using activated silica gels. The phytochemical analysis was adapted from Anning's thesis work [12], which showed the presence of alkaloids, flavonoids, saponins, tannins, steroids and anthraquinone. Glycosides and triterpenoids were not detected in the extract.

### Extract preparation

A 500 μg ml^−1^ stock of ethanolic leaves extract of *E. ivorense* was dissolved in 1 % DMSO in M199 complete solution. A serial dilution of 250, 125, 62.5, 31.3 and 15.6 µg ml^−1^ of the stock was made with M199 medium. A 2.5 μg ml^−1^ of amphotericin B was also prepared as a positive control drug. The preparation was stored at 4 °C for 7 days [12, 13].

### *Leishmania* parasites' culture

Cryopreserved *Leishmania* promastigotes of Ghanaian *Leishmania* isolate and *L. donovani* were rapidly thawed and cultured in M199 complete medium containing 10 % of foetal bovine serum (FBS), 1 % basal medium Eagle (BME), vitamins, and 0.25 % gentamicin with 1 % of urine and cultured at 25 °C in an incubator [27]. The motility of the promastigotes was monitored daily for 72 h under the X40 inverted microscope and its viability determined by staining the live parasites with the trypan blue dye for differential count and exclusion of dead cells from the live-cell parasites [28]. The promastigotes counts were made daily by estimating the number of parasites using a haemocytometer. Large cultures of the promastigotes were made for extract sensitivity tests on the parasites.

### Extract sensitivity test and trypan blue quantification assay

The number of promastigotes of *Leishmania* isolates from Ghana and *L. donovani* were adjusted to 1×106 cell ml^−1^ and seeded into 96-well plates in triplicates for each of the serialized concentration (500, 250, 125, 62.5, 31.3 and 15.6 µg ml^−1^) of the extracts. In total, 2.5 µg ml^−1^ of standard amphotericin B and 1 % DMSO (dimethyl sulfoxide) were used as positive and negative drug controls, respectively. Parasites were cultured at 25 °C for 24 h. The efficient extract concentration for promastigote growth inhibition was estimated using 0.4 % trypan blue dye with 1 : 1 dilution of the cell suspension, incubated in a humid chamber for 10 min, followed cell counting by a haemocytometer and determination of viability [29, 30]. Promastigote inhibition by serially diluted concentrations of the extract was quantified by measuring the intensity of trypan blue by a spectrophotometer [31]. The 62.5 µg ml^−1^ threshold of the extract concentration showed to have the best sensitivity and specificity to inhibit the promastigote of the Ghanaian *Leishmania* isolate. Promastigote inhibition was measured at 12, 24, 48 and 72 h. To confirm 62.5 µg ml^−1^ as the best extract concentration for promastigote inhibition, we tested for 12 h inhibition.

For the trypan blue quantification assay, the extract-treated promastigotes were stained with 0.4 % trypan blue solution at room temperature for 10 min followed by thorough but gentle washing three times with 200 μl of culture medium to remove the stain solution and background by rocking and centrifuging at 1000 r.p.m. for 3 min. Randomly, some of the samples were microscopically observed to check the ratio of dead to living promastigotes before lysing the samples with 200 μl of 1 % sodium dodecyl sulfate (SDS) in a complete culture medium. To control the background trypan blue intensity, empty tubes were incubated with trypan blue and treated in a similar way to the test samples and used as a sham control. Also, samples from the log phase of promastigotes were also lysed and used as a control for viable promastigotes. A 72 h culture of *Leishmania* isolates in 2.5 μg ml^−1^ of amphotericin B, which kills more than 90 % of promastigotes, were used as an inhibition control for extract inhibition. Samples were analysed spectrophotometrically at 590 nm using a microtitre plate reader (800TM TS absorbance reader, BioTeK instrument, USA). The mean absorbance values obtained were controlled for the background absorbance [31]. The promastigote inhibition by the extract was expressed as a percentage of live promastigotes, prepared at specific time points of 12, 24, 48 and 72 h.

### Data analysis

The data obtained were recorded using a Microsoft Excel 2010 worksheet and analysed with SPSS version 16. The promastigote inhibition by the extract and amphotericin B were quantified using a light microscope and spectrophotometer. The results were expressed as the percentage mean inhibition of the promastigotes. The effective extract concentration of *E. ivorense* was estimated using concentration cut-off value statistics and the receiver operating characteristic (ROC) curve. Time- and concentration-dependent inhibition of the concentration thresholds of the extract were calculated using mean±se, Huber’s estimator and a control chart. Monte Carlo chi-square was used to compare inhibition activity against the MIC of *E. ivorense* extract and amphotericin B against *L. donovani* and *L. enriettii* complex isolates.

## Results

### Selection of threshold concentration of ethanolic leaf extract of *E. ivorense* for optimum inhibition of the promastigote stage of *L. enriettii* complex isolates from Ghana

**Fig. 1.**
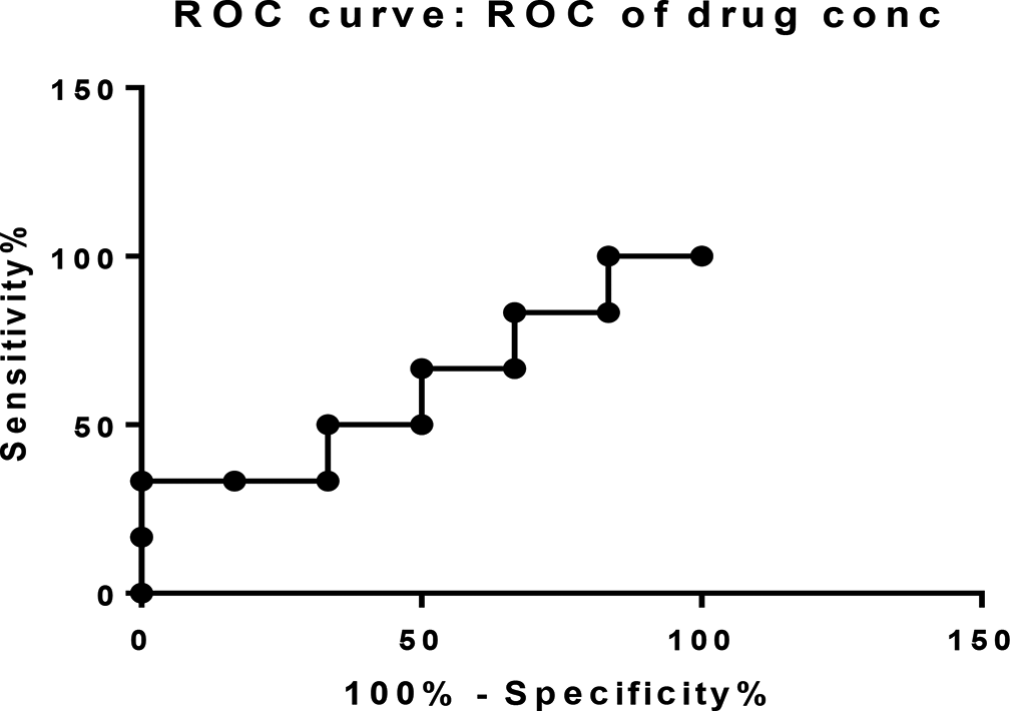
Receiver operating characteristic curve (ROC) showing the sensitivity and specificity of the concentration cut offs of the *E. ivorense* leaf extract on *L. enriettii* promastigote inhibition.

The threshold of *E. ivorense* extract to inhibit the promastigote stage of the *L. enriettii* complex isolate was selected by exposing the promastigotes to a serial dilution of the extract (15.6, 31.2, 62.3, 125, 250 and 500 μg ml^−1^) for 24 h. The inhibition of the promastigotes was measured after 24 h of post-extract exposure. The viable promastigote cells were detected by staining with trypan blue stains and absorbance measured at 590 nm using a spectrophotometer [32]. The MIC for the serially diluted extracts against the promastigote *L. enriettii* complex isolates were determined using concentration cut-off statistics. The results showed that concentration between >53 μg ml^−1^ (% sensitivity of 66.67, 95 % CI [22.28–95.67] with % specificity of 33.33, 95 % CI [4.327–77.72]; likelihood ratio of 1.000) and >64.5 μg ml^−1^ (% sensitivity of 66.67, 95 % CI [22.28–95.6] with % specificity of 50.00, 95 % CI [11.81–88.19]; likelihood ratio of 1.333) had the MIC of the ethanolic leaf extract of *E. ivorense* required to significantly inhibit the growth of the promastigote *L. enriettii* complex isolate from Ghana. Although extract concentration >215.5 μg ml^−1^ showed 100 % specificity, 95 % CI (54.07–100.0), it recorded the lowest sensitivity of 16.67 %, 95 % CI (0.4211–64.12). Again, concentration >15 μg ml^−1^ recorded 100 % sensitivity, 95 % CI (54.07–100.0) with lowest specificity of 16.67 %, 95 % CI (0.4211–64.12) with a likelihood ratio of 1.200 (Table 1). Similarly, the area under the curve of ethanolic leaf extract of *E. ivorense* with a concentration between >53 μg ml^−1^ and >64.5 μg ml^−1^ showed MIC with 50 % sensitivity for inhibiting the growth of the promastigote stage of *L. enriettii* complex isolates from Ghana in *in vitro* culture (Fig. 1). This indicates that the MIC of the *E. ivorense* extract that can significantly inhibit the *L. enriettii* complex isolate upon exposure is 62.3 μg ml^−1^.

**Table 1.** The sensitivity and specificity of the concentration cutoffs of the *E. ivorense* leaf extract on *L. enreittii* promastigote inhibition.

Conc. cut off (µg ml^−1^)	% sensitivity (95 % CI)	% specificity (95 % CI)	Likelihood ratio
15.00	100.0 (54.07–100.0)	16.67 (0.4211–64.12)	1.200
30.50	83.33 (35.88–99.58)	16.67 (0.4211–64.12)	1.000
44.50	83.33 (35.88–99.58)	33.33 (4.327–77.72)	1.250
53.00	66.67 (22.28–95.67)	33.33 (4.327–77.72)	1.000
64.50	66.67 (22.28–95.6)	50.00 (11.81–88.19)	1.333
82.50	50.00 (11.81–88.19)	50.00 (11.81–88.19)	1.000
99.00	50.00 (11.81–88.19)	66.67 (22.28–95.6)	1.500
108.0	33.33 (4.327–77.72)	66.67 (22.28–95.6)	1.000
133.5	33.33 (4.327–77.72)	83.33 (35.88–99.58)	2.000
166.5	33.33 (4.327–77.72)	100.0 (54.07–100.0)	
215.5	16.67 (0.4211–64.12)	100.0 (54.07–100.0)	

CI, confidencet interval.

### Time-dependent growth inhibitory activity of 62.3 μg ml^−1^ of ethanolic leaf extract of *E. ivorense* against the promastigote stage of the *L. enriettii* complex isolate

**Fig. 2.**
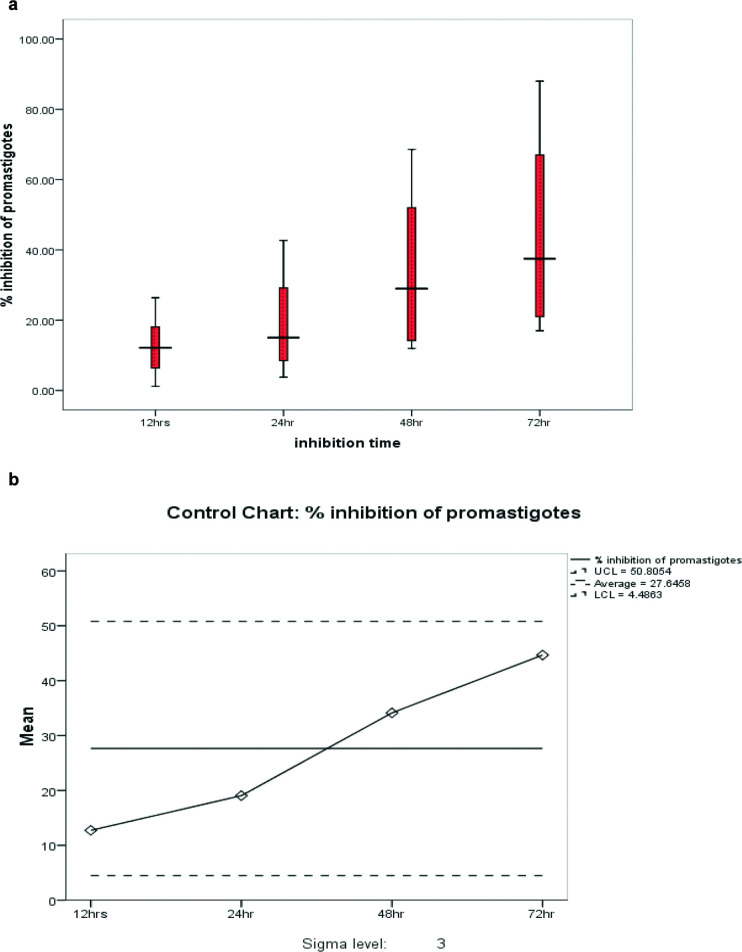
Inhibition of *L. enriettii* promastigote at 62.3 μg ml^−1^ of *E. ivorense* leaf extract. (a) Box plot showing inhibition of *L. enriettii* promastigotes per time of the *E*. *ivorense* leaf extract treatment. (b) Control chart for *L. enriettii* promastigote inhibition per time of the *E. ivorense* leaf extract treatment.

**Fig. 3.**
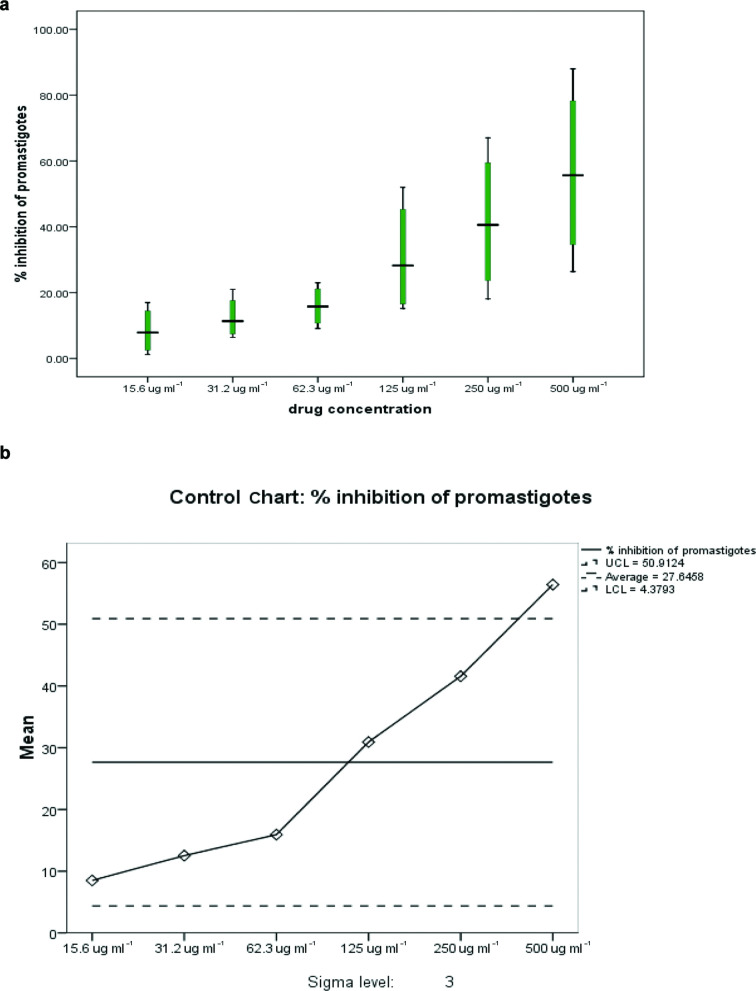
Inhibition of *L. enriettii* promastigotes per the serially diluted concentration of the *E. ivorense* leaf extract treatment. (a) Box plot showing promastigote inhibition per the concentration of *E. ivorense* leaf extract. (b) Control chart for promastigote inhibition per concentration of the *E. ivorense* leaf extract.

The effectiveness of 62.3 μg ml^−1^ of *E. ivorense* extract to inhibit the growth of *L. enriettii* complex isolate promastigotes was tested by exposing the parasites to the extract and the inhibitory effect measured at 12, 24, 48 and 72 h. The highest promastigote inhibition with 62.3 μg ml^−1^ was observed at 72 h with the mean inhibition of 44.67 % (standard error of the mean=11.83 %), 95 % CI (14.16–75.17 %); Huber’s estimator=40.58 while the lowest promastigote inhibition was observed at 12 h post-treatment (mean inhibition=12.73 %; standard error of the mean=3.69 %; 95 % CI [3.25–22.21 %] and Huber’s estimator=12.18) (Table 2). The effectiveness of 62.3 μg ml^−1^ of the extract to inhibit the promastigote stage of the *L. enriettii* complex isolate was assessed using a univariate control chart. The result confirmed the time-dependent inhibitory activity of 62.3 μg ml^−1^ of the extract against the promastigotes of the *L. enriettii* complex isolate. The lowest inhibition was observed at 12 h post-treatment and the highest promastigote inhibition was observed at 72 h, as shown in the box plot (Fig. 2a). The quality of promastigote inhibition by 62.3 μg ml^−1^ over time showed that promastigote inhibition falls between the upper control limit (UCL) of 50.8054 and lower control limit (LCL) of 4.4863 with an average of 27.6458 (Fig. 2b). This indicates that *E. ivorense* extract concentration of 62.3 μg ml^−1^ falls into the desired sensitivity and specificity obtained from the concentration cut-off values (Table 1) and has an inhibitory activity against the promastigote stage *L. enriettii* complex isolate. Again, high concentrations of *E. ivorense* extract showed a strong inhibitory effect on promastigotes of the *L. enriettii* complex isolate at 125 (mean inhibition=30.93 %; standard error of the mean=8.78 %; 95 % CI [2.99–58.85 %] and Huber’s estimator=29.11), 250 and 500 μg ml^−1^ (mean inhibition=56.43 %; standard error of the mean=13.65 %; 95 % CI [12.99–99.86 %] and Huber’s estimator=55.65) at 24 h post-treatment (Table 3). Similarly, the univariate control chart also showed that the higher the concentration, the greater the inhibition means of the promastigotes. However, 500 μg ml^−1^ of *E. ivorense* extract crossed the UCL of 50.912 suggesting potential toxicity (Fig. 3a, b). This is in agreement with the concentration cut-off analysis, which showed high percentages of specificity but low percentages of sensitivity to *L. enriettii* isolate promastigote inhibition (Table 1).

**Table 2.** Time-dependent inhibition of *L. enriettii* promastigotes treated with the optimal concentration of 62.3 μg ml^−1^ of the *E. ivorense* leaf extract

	% inhibition per time (cell ml^−1^)		
Time hr^−1^	mean±se	95 % CI	Huber’s estimator
12	12.73±3.69	3.25–22.21	12.18
24	19.05±5.63	3.81–34.28	16.79
48	34.13±9.37	10.04–58.23	31.49
72	44.67±11.87	14.16–75.17	40.58

S.E, Standard error of the mean; CI, confidencet interval.

**Table 3.** Inhibition of *L. enriettii* promastigotes treated with serially diluted concentration of the *E. ivorense* leaf extract

	% inhibition per conc. (cell ml^−1^)		
Conc. µg ml^−1^	Mean±se	95 % CI	Huber’s estimator
15.6	8.50±3.65	−3.12–20.12	8.07
31.2	12.53±3.27	2.12–22.93	11.44
62.3	15.93±3.18	5.81–26.04	15.83
125	30.93±8.78	2.99–58.85	29.11
250	41.58±11.03	6.48–76.67	40.65
500	56.43±13.65	12.99–99.86	55.65

S.E, Standard error of the mean; CI, confidencet interval.

The effect of *E. ivorense* leaf extract was tested with a standard leishmanicidal drug amphotericin B (2.5 μg ml^−1^) after 72 h post-treatment using *L. donovani* as a control parasite. The result showed that there was no significant inhibitory effect between the *E. ivorense* extract and amphotericin B on *L. donovani* (χ^2^=1.012, Monte Carlo statistics=0.356, 95 % CI [0.344–0.369]). However, *E. ivorense* extract significantly inhibited the promastigotes of the *L. enriettii* isolate from Ghana comparable to amphotericin B (χ^2^=15.934, Monte Carlo statistics=0.00, 95 % CI [0.00–0.00]) (Table 4). This suggests that 62.3 μg ml^−1^ of *E. ivorense* leaf extract can be used as the best concentration for the treatment of *L. enriettii* complex isolates from Ghana.

**Table 4.** Comparable inhibitory effect of the optimized concentration of the *E. ivorense* leaf extract and amphotericin B against the *L. enriettii* complex isolate from Ghana and *L. donovani*

	Cell ml^−1^ (% inhibition of promastigotes)			
	*E. ivorense*	Amphotericin B	×^2^	Monte Carlo (99 % CI)
*L. donovani*	77 (12.38)	90 (24.67)	1.012	0.356 (0.344–0.369)
*L. enriettii* complex	174 (36.42)	258 (42.81)	15.934**	0.00 (0.00–0.00)

* Significant (*P*<0.05); ** Significant (*P*<0.01); *** Highly significant (*P*<0.001).

## Discussion

The isolation of the *L. enriettii* complex isotype in the Volta Region of Ghana is an indication of the expansion, emergence and adaptation of zoonotic leishmaniasis [24, 33, 34]. This isolate causes cutaneous leishmaniasis [24]. Understanding of the biology of this new isolate is important for efficient treatment and eradication of cutaneous leishmaniasis from Ghana. The infectivity of the *L. enriettii* complex isolate requires the understanding of the amastigote stage of the parasite. Current research findings indicate that wild guinea pigs are the host of the *L. enriettii* complex, which suggests that the isolate can be adapted into a guinea-pig model. As part of the preparatory laboratory adaptation of the isolate, the MIC of crude leaf extracts of *E. ivorense* with leishmanicidal activity was determined. The extract of *E. ivorense* is affordable, accessible and it has shown efficient antileishmanial activity against the promastigote stage of this *L. enriettii* complex isolate [12, 13]. The extracts of *E. ivorense* have been shown to have lower side effects on the host organisms [35, 36].

Identification of a potential therapeutic agent for the treatment of extensive hyper-immune stimulation underlying cutaneous leishmaniasis such as diffuse CL or Post kala-azar dermal leishmaniasis (PKDL) that result in disfiguring and disability is essential for the treatment of *L. enriettii* complex infection [37, 38]. These CL-mediated immune dysfunctions make *E. ivorense* extracts a therapeutic agent of choice aside its leishmanicidal activities against the promastigote stage of the *L. enriettii* complex isolate [39, 40]. The anti-inflammatory, antiallergic, wound-healing activities of *E. ivorense* makes it a potential desired drug candidate for the topical treatment of cutaneous leishmaniasis [41].

Here we show that 62.3 μg ml^−1^ as the MIC of *E. ivorense* leaf extract has the best specificity and sensitivity for its leishmanicidal effects against the promastigote stage of the *L. enriettii* complex isolate from Ghana. Many antimonial drugs eliminate *Leishmania* parasites through immunomodulation mechanisms [42]. For instance, miltefosine activates host pro-inflammatory chemokines such as IFN-γ, TNF-α and IL-12 while upregulating CCL2, CXCL-9 and CXCL-10 [43–46]. The immune regulatory activities of *E. ivorense* extract underlying its anti-inflammatory, antiallergic and wound-healing abilities could facilitate rapid treatment of cutaneous leishmaniasis [39, 41]. Although the mechanisms underlying the anti-inflammatory, antiallergic and the wound-healing abilities of *E. ivorense* are not well understood, yet the immune regulation coupled with its leishmanicidal ability can be capitalized on for the treatment of cutaneous leishmaniasis.

The adaptation of *L. enriettii* complex isolates from Ghana into a guinea-pig model is essential for the study of the biology of this geographical *Leishmania* isolate. This is important because of the variation in drug sensitivity and efficacy among species and geographical *Leishmania* isolates [47]. The genetic composition and biochemical characteristics among the species had been the cause of variation in drug sensitivity to different species of *Leishmania* [48]. For instance, *Leishmania* isolates from Sudan and Ethiopia show 14.3 and 93.1 % cure rate by paromomycin treatment; dithiocarbamates and its structurally related compounds have good efficacy against intracellular *L. donovani* and *L. major* but are ineffective against intracellular *L. amazonensis,* whereas miltefosine exhibits species-specific susceptibility [49, 50].

In conclusion, 62.5 μg ml^−1^ of *E. ivorense* leaf extract is the MIC with the best leishmanicidal activities against the promastigote stage of the *L. enriettii* complex isolate from Ghana. This suggests that we can capitalize on its immunomodulatory effects against inflammation, and wound-healing effects as a drug candidate of choice for topical treatment of cutaneous leishmaniasis caused by the *L. enriettii* complex isolate.
